# Characterization and applications of Nanobodies against human procalcitonin selected from a novel naïve Nanobody phage display library

**DOI:** 10.1186/s12951-015-0091-7

**Published:** 2015-05-06

**Authors:** Junrong Yan, Pingyan Wang, Min Zhu, Guanghui Li, Ema Romão, Sheng Xiong, Yakun Wan

**Affiliations:** The Key Laboratory of Developmental Genes and Human Disease, Ministry of Education, Institute of Life Sciences, Southeast University, Nanjing, 210096 PR China; Jiangsu Nanobody Engineering and Research Center, Nantong, 226010 PR China; Institute of Biomedicine & National Engineering Research Center of Genetic Medicine, College of Life Science and Technology, Jinan University, Guangzhou, 510630 PR China; Laboratory of Cellular and Molecular Immunology, Vrije Universiteit Brussel, Faculty of Science, Pleinlaan 2, 1050, Brussels, Belgium

**Keywords:** Bactrian camel, VHH, Naïve phage-displayed library, Human procalcitonin, Biotin-Streptavidin-System, Sandwich ELISA

## Abstract

**Background:**

Nanobodies (Nbs) are single-domain antigen-binding fragments derived from the camelids heavy-chain only antibodies (HCAbs). Their unique advantageous properties make Nbs highly attractive in various applications. The general approach to obtain Nbs is to isolate them from immune libraries by phage display technology. However, it is unfeasible when the antigens are toxic, lethal, transmissible or of low immunogenicity. Naïve libraries could be an alternative way to solve the above problems.

**Results:**

We constructed a large camel naïve phage display Nanobody (Nb) library with great diversity. The generated library contains to 6.86 × 10^11^ clones and to our best of knowledge, this is the biggest naïve phage display Nb library. Then Nbs against human procalcitonin (PCT) were isolated from this library. These Nbs showed comparable affinity and antigen-binding thermostability at 37°C and 60°C compared to the PCT Nbs from an immune phage-displayed library. Furthermore, two PCT Nbs that recognize unique epitopes on PCT have been successfully applied to develop a sandwich enzyme-linked immunosorbent assay (ELISA) to detect PCT, which showed a linear working range from 10-1000 ng/mL of PCT.

**Conclusion:**

We have constructed a large and diverse naïve phage display Nb library, which potentially functioning as a good resource for selecting antigen-binders with high quality. Moreover, functional Nbs against PCT were successfully characterized and applied, providing great values on medical application.

**Electronic supplementary material:**

The online version of this article (doi:10.1186/s12951-015-0091-7) contains supplementary material, which is available to authorized users.

## Background

Since their discovery, antibodies have become excellent candidates for research, diagnostic and therapeutic applications due to their high affinity and specificity characteristics [1,2]. Most applications rely on conventional immunoglobulin G (IgG) molecules. These antibodies, produced by B lymphocytes, comprise two identical heavy chains and two identical light chains [3]. The molecular weight of an IgG is approximately 160 kDa, and it is a complex molecule consisting of heavy and light chains, which together form the antigen binding site [4]. However, some limitations of the IgG, such as its large size and complicated structure, the time consuming and costly production, unstable behaviors and their reliance on mammalian cells expression, [5] create numerous problems on practical applications. An alternative approach is to use some antibody fragments, such as antigen-binding fragments (Fabs) and single-chain variable fragments (scFvs).

With the rapid development of molecular gene engineering techniques, Fabs (50 kDa) and scFvs (27 kDa) have been widely used as alternatives to classic antibodies. These antibody fragments can maintain the specificity to antigens. Fabs are the oldest class of monoclonal antibody derived fragments, which are clearly thoroughly explored [6]. Unfortunately, due to their relatively large size of approximately 50 kDa, Fabs yield large oligomeric molecules [7]. scFvs are the smallest intact antigen-binding fragments that can be derived from a conventional IgG molecule [4]. They are recombinant antigen-binding fragments in which the variable regions of light and heavy chains are combined into a single polypeptide. The small size of scFvs permits penetration into obstructed locations that inaccessible for full-size conventional antibodies, such as tumors [8]. However, scFvs tend to form bivalent or higher oligomers [7]. Additionally, the production of these fragments is difficult due to their low yield, poor solubility and stability [9]. Moreover, their susceptibility to temperature and pH [10] make them unsuitable for use in biosensors as diagnostics or as therapeutic candidates.

Aside from classic IgGs, researchers found that certain animals, such as camelids and sharks, [11,12] are able to produce a unique kind of antibody naturally lacking light chains, termed heavy chain-only antibodies (HCAbs). The antigen-binding sites of the HCAbs are composed of two single variable domains, named the variable domain of heavy chain of the heavy chain-only antibody (VHH) [4]. VHHs are distinguished from the conventional IgG and various IgG derivatives by their unique properties of molecular size (2.5 nm in diameter and 4 nm in height), [13] therefore, it is also known as Nanobody® (Nb). Nanobodies (Nbs) are the smallest fully functional antigen-binding fragments [13]. Due to their small size (15 kDa) and single-domain nature, Nbs are ideal entities for basic research, biosensor and therapeutic applications. This monomeric antigen-binding fragment shows several special features in terms of their high thermal and conformational stability, [14] high aqueous solubility, straightforward production in micro-organisms, [9] resistance to acid and alkaline pH, [15] excellent affinity and specificity, [13] ease of manufacture [7] and low immunogenicity. In addition, Nbs exhibit superior body distribution, tissue penetration and faster blood clearance rate [9]. These advantageous properties make Nbs attractive and valuable tools for various applications.

Generally, Nbs can be isolated from an immune Nb library. VHHs genes can be reverse-transcribed from the mRNA of peripheral blood lymphocytes (PBLs) of animals that were immunized with the antigens of interest. Phage display technology is the most popular method to isolate antigen-binding clones [16]. Although camels, llamas and dromedaries have all been immunized to produce antigen binders in many studies, [17-19] this routine way is often unrealistic when the antigens are toxic, lethal, transmissible, of low immunogenicity, or even nonimmunogenic small molecular compounds [16,20]. In addition, the immunization process is time-consuming and normally takes months to raise a satisfactorily immune response [16]. Alternatively, naïve phage display libraries could solve the above problems. Non-immunized camels can be used to generate unbiased or non-immune libraries, from which antigen binders of interest can be selected.

In this study, based on the VHHs genes, which were amplified from the mRNA of peripheral blood lymphocytes (PBLs) of twenty four non-immunized bactrian camels and a spleen from another non-immunized bactrian camel, we successfully constructed a large naïve phage-displayed library with great diversity. The library contains 6.86 × 10^11^ clones, with an insertion rate of clones having a VHH of nearly 100%. Then Nbs against human procalcitonin (PCT) were isolated from this phage-displayed library. PCT is a highly valuable diagnostic biomarker of bacterial infections, which is frequently used as a reliable marker to support the risk evaluation of seriously sick people progressing to severe sepsis and septic shock [21,22]. Furthermore, by comparing the PCT Nbs from an immune phage-displayed library constructed in our previous study, [23] the naïve library derived PCT Nbs showed comparable affinity and antigen-binding thermostability at 37°C and 60°C. In addition, two Nbs recognizing unique epitopes of PCT, were used to develop a sandwich enzyme-linked immunosorbent assay (ELISA) to detect the bacterial protein. Consequently, the Nbs from the naive library were successfully used for capture and detection of native PCT in the ELISA based diagnostic assay allowing the sensitive measurement of the disease marker. Altogether, such a large and diverse naïve phage-displayed library represents a good resource for selecting functional binders with high affinity and specificity, hence being an essential alternative to immune libraries.

## Results and discussion

### Construction of a naïve VHH library

**Figure 1:**
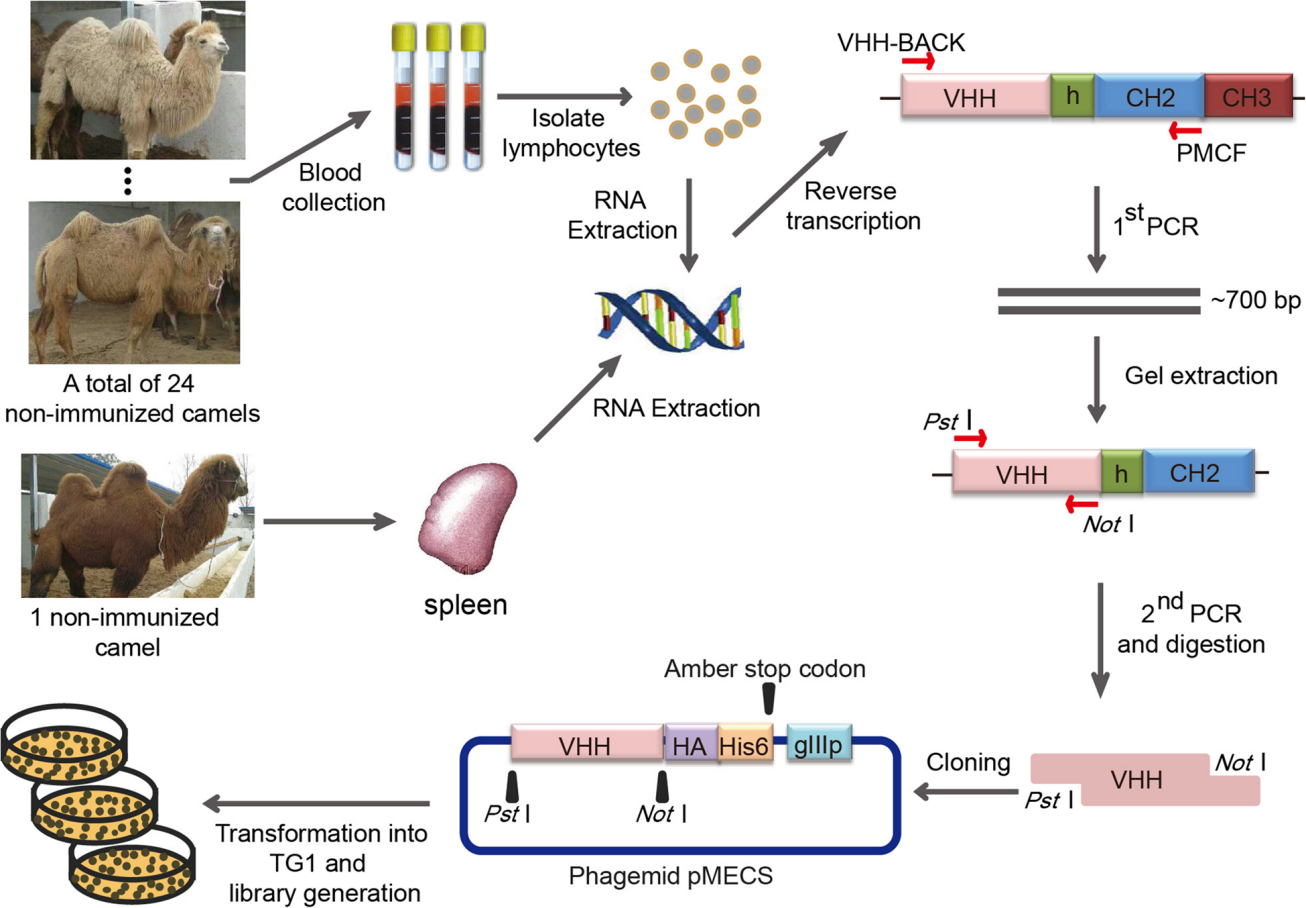
Scheme of strategy to construct the naïve library.

**Figure 2:**
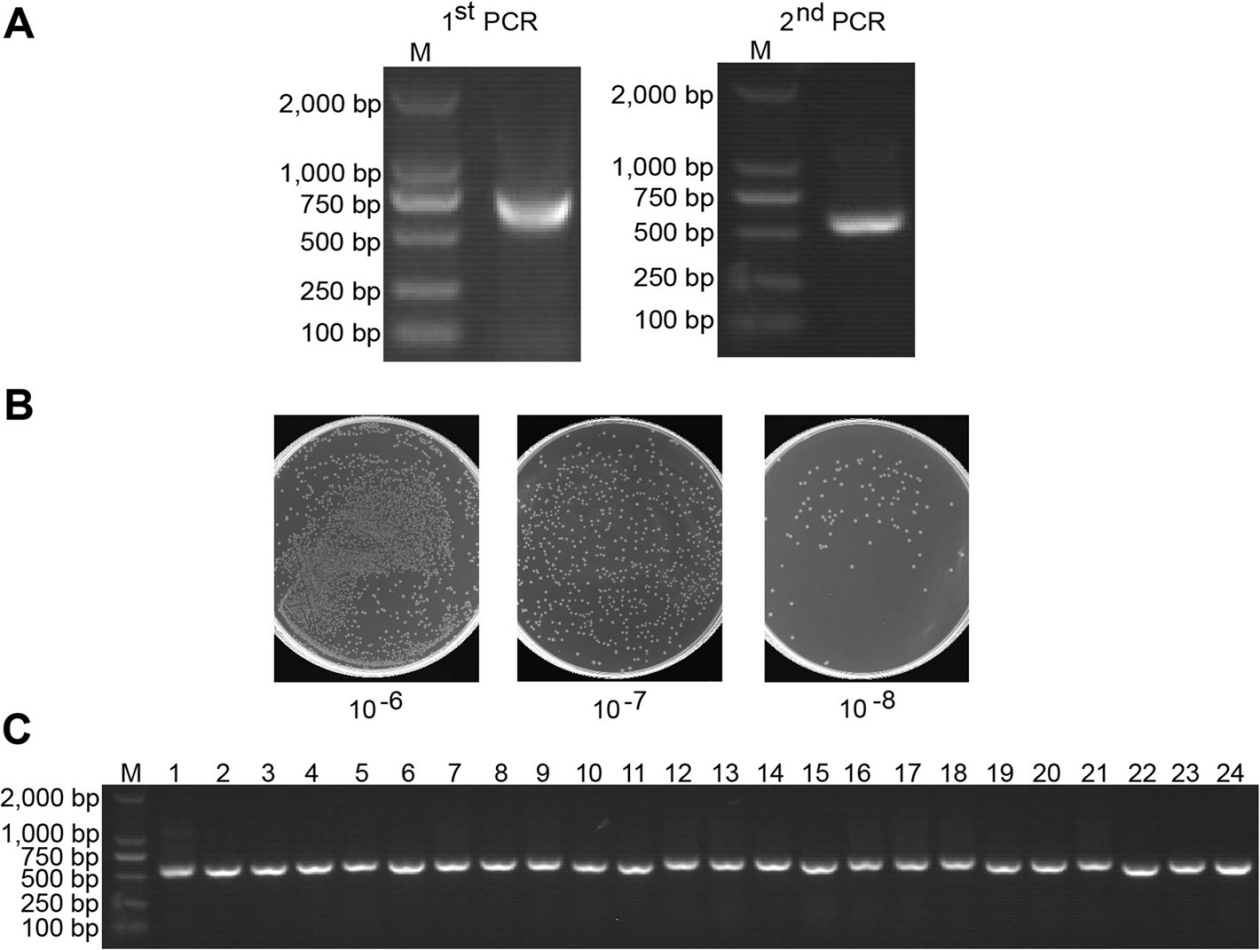
Construction of the naïve library. **(A)**The VHH genes were obtained by two steps PCR. **(B)** The library size was measured by counting the colonies number after serial dilution. **(C)** 24 colonies were randomly picked to estimate the correct insertion rate of VHH genes by PCR amplification.

The general way to obtain Nbs is screening the antigen-specific Nbs from an immune library. However, when the antigens are toxic, low immunogenic, or nonimmunogenic small molecular compounds, immunization will not be practical. Such circumstances also impose great challenges for conventional antibody generation. Additionally, the process takes more than one month for camels to raise a sufficiently high immune response. Isolating Nbs from a naïve library could be an alternative way. To construct a large and highly diverse naïve Nb library, as shown in Figure 1, total RNA was isolated from PBLs collected from twenty four non-immunized bactrian camels and a spleen from another non-immunized camel. Amplicons spanning the VHH-CH2 exons with a size of 700 bp were generated during the primary PCR (Figure 2A). The VHH encoding gene fragments were then amplified by the second PCR (Figure 2B) with the 700 bp fragment purified with gel extraction as the template using degenerated primers that introduced the *Pst* I and *Not* I restriction sites (Figure 1). The *Pst* I and *Not* I double-digested amplicons were cloned into the phagemid vector pMECS allowing the expression of C-terminal Hemagglutinin (HA)-His6-tagged Nbs, after that, the recombinant vector was electro-transformed into competent TG1 cells. Dilution plating of the cultured library indicated a total size of 6.86 × 10^11^ colony-forming units (CFU) (Figure 2C), being the largest phage-displayed Nb library to our understanding. PCR analysis of 24 randomly picked clones demonstrated a frequency of clones having a complete VHH insert of 100% (Figure 2D), which meant the functional capacity of the library was very high. The sequencing results of the 300 randomly picked clones showed the library has a high diversity, 9 groups containing 20 clones exhibited the same sequence in CDR3 regions with each other within the groups, and among them, 2 groups containing 4 clones showed the exactly same amino acids all through the whole VHH with each other within the groups. Thus, there are 289 kinds of different amino acids sequences in CDR3 regions among these 300 clones (Additional file 1: Figure S1-S10), which indicated the diversity was high. Overall, we have successfully constructed a naïve phage display Nb library of high quality for the following selection of Nbs against PCT.

### Selection of PCT Nbs

**Figure 3:**
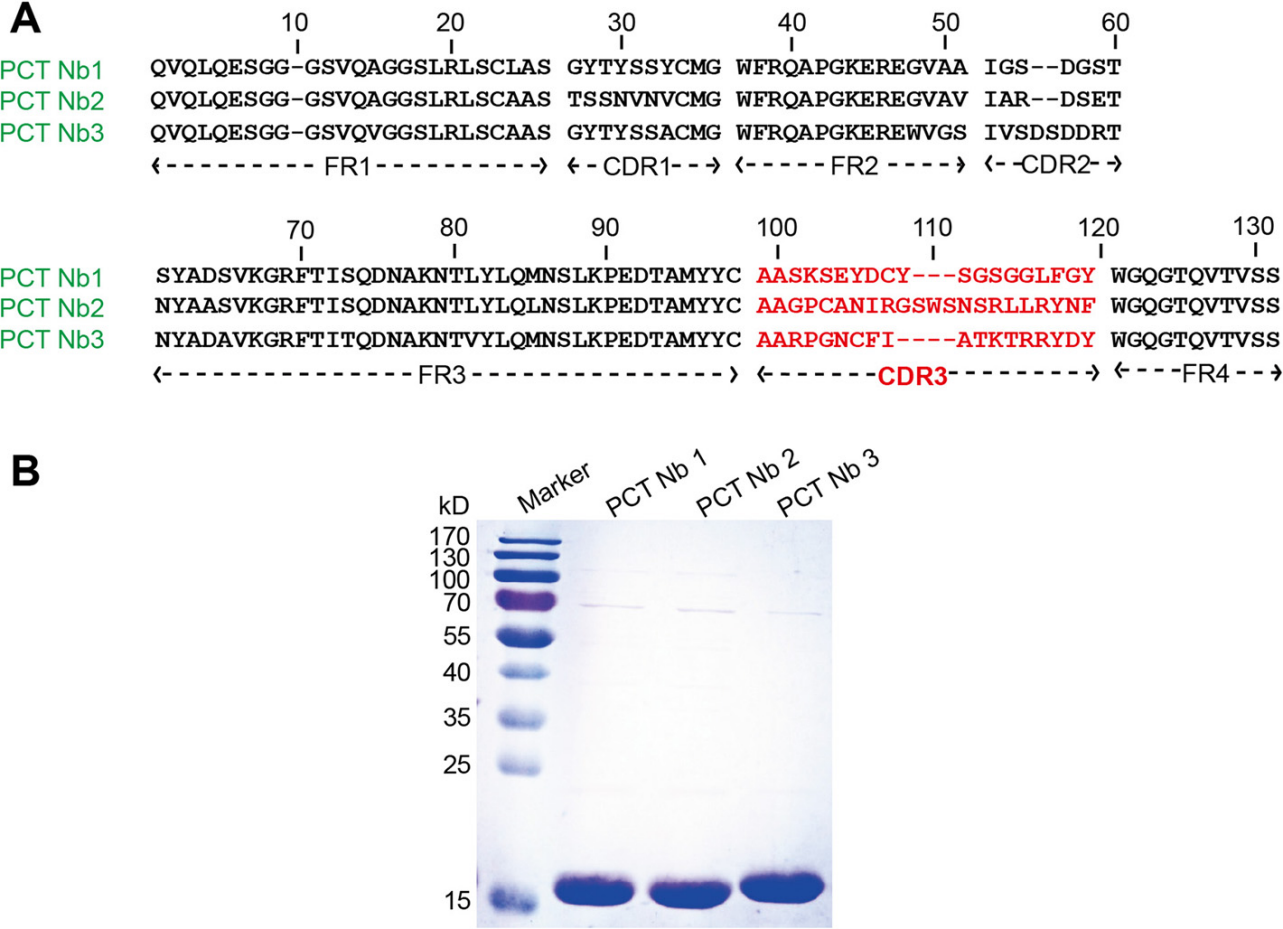
Non-immune library derived PCT Nbs. **(A)** Amino acid sequence alignment of three PCT-specific Nb families as classified by CDR3. Amino acids positions of the framework region (FR) and of the three antigen-binding loops (CDR1, CDR2 and CDR3) are numbered according to the IMGT Scientific chart for the V-Domain and are indicated at bottom. **(B)** SDS-PAGE analysis of purified Nbs.

By taking advantage of the high diversity of the naïve Nb library, we identified PCT Nbs by bio-panning to validate the quality of the library using phage display technology. As it is a very large library, we only took a small sample of the library to investigate whether good quality of binders can be isolated as a previous study did [24]. Although we did not screen the whole naïve library herein and it may lose the diversity of Nbs during selection, we were still able to retrieve PCT Nbs with comparable characteristics to those of the immune PCT Nbs according to the following results in this study, which in turn shows that the naïve library can be indeed a powerful and resourceful alternative to the immune libraries. During the screening, we calculated the relative enriching efficiency of phage particles eluted from wells coated with PCT versus those without antigens. Next, 95 individual colonies were randomly picked to identify specific Nbs by performing periplasmic extraction ELISA (PE-ELISA) after the 4^th^ round of panning. A total of 34 positive colonies with a binding ratio of more than 3 were identified. The positive colonies were sent for sequencing, and seven different sequences were obtained. Finally, the PCT-specific Nbs were classified into three families based on the diversity of their amino acid sequences in complementarity determining region (CDR) 3 (Figure 3A). These Nbs were named PCT Nb1, Nb2 and Nb3. In our previous study, we have obtained several anti-PCT Nbs from an immune library, which were successfully used to develop a Nb-based electrochemiluminescent immunosensor for sensitive detection of human PCT [23]. The amino acids sequences of CDR3 comparison between the two different libraries-derived PCT Nbs demonstrates that they do not share any sequence similarity. Thus far, we have successfully isolated diverse Nbs, indicating that the naïve library is a great resource for Nbs selection. Moreover, we need to further assess the quality of the non-immune library by screening more antigens.

### Expression and purification of soluble Nbs

In order to express the isolated Nbs, we transformed the recombinant phagemid from *E. coli* TG1 to WK6 cells. TG1 cells suppress the amber stop codon between VHH and the gene III in phagemid pMECS, resulting in the expression of VHH-gene III fusions. However, WK6 is a non-suppressor strain and will not express the gene III fusion, but only the VHH, which contains at its C-terminal end a HA-His6-tag. (Figure 3B). Soluble Nbs were extracted from the periplasm by osmotic shock and they were purified on a NI-NTA Superflow Sepharose column using 500 mM imidazole for elution. SDS-PAGE analysis demonstrated the good quality of Nbs obtained with high purity (Figure 3B) and milligram quantities of production were yielded were yielded from 1 L of culture (Table 1).

**Table 1:** **Properties of PCT Nbs from the naïve library and the immune library** [23]

**VHH**	**M**_**W**_^**a**^ **(kDa)**	**pI** ^**b**^	**ε** ^**c**^ **(g/L)**	**Yield (mg/L)**	**K**_**on**_ **(M** ^**-1**^ **s** ^**-1**^ **)**	**K**_**Off**_ **(s** ^**-1**^ **)**	**K**_**D**_ **(M)**
PCT Nb1	15.47	6.03	1.964	7.5	4.16 × 10^4^	5.10 × 10^-4^	1.23 × 10^-8^
PCT Nb2	15.18	8.73	1.833	4.5	1.47 × 10^5^	1.97 × 10^-4^	1.34 × 10^-9^
PCT Nb3	16.08	7.19	1.954	6.0	4.31 × 10^4^	3.83 × 10^-4^	8.89 × 10^-9^
PCT Nb20	16.05	6.13	2.498	7.1	2.85 × 10^4^	9.52 × 10^-4^	3.34 × 10^-8^
PCT Nb45	15.71	7.95	2.027	4.9	1.64 × 10^3^	4.02 × 10^-5^	2.45 × 10^-8^
PCT Nb50	16.12	7.17	2.577	8.7	2.39 × 10^4^	6.34 × 10^-4^	2.65 × 10^-9^
PCT Nb52	15.59	6.70	2.367	9.3	2.32 × 10^4^	6.10 × 10^-4^	2.63 × 10^-8^
PCT Nb85	16.07	7.17	2.578	6.4	2.11 × 10^4^	8.21 × 10^-4^	3.89 × 10^-9^
PCT Nb86	16.18	8.36	2.317	5.5	2.52 × 10^4^	8.65 × 10^-4^	3.43 × 10^-9^
PCT Nb87	16.64	6.38	1.825	10.3	4.18 × 10^5^	2.58 × 10^-4^	6.17 × 10^-9^
PCT Nb88	15.63	8.80	1.925	7.6	1.99 × 10^4^	8.05 × 10^-4^	4.04 × 10^-9^

^a^Molecular weight (Mw) includes HA and His_6_ tag.

^a,b,c^Theoretical isoelectric point (pI), Mw and extinction coefficient (ε) were calculated by the ExPAsy ProtParam Tool.

### Binding thermostability analysis

**Figure 4:**
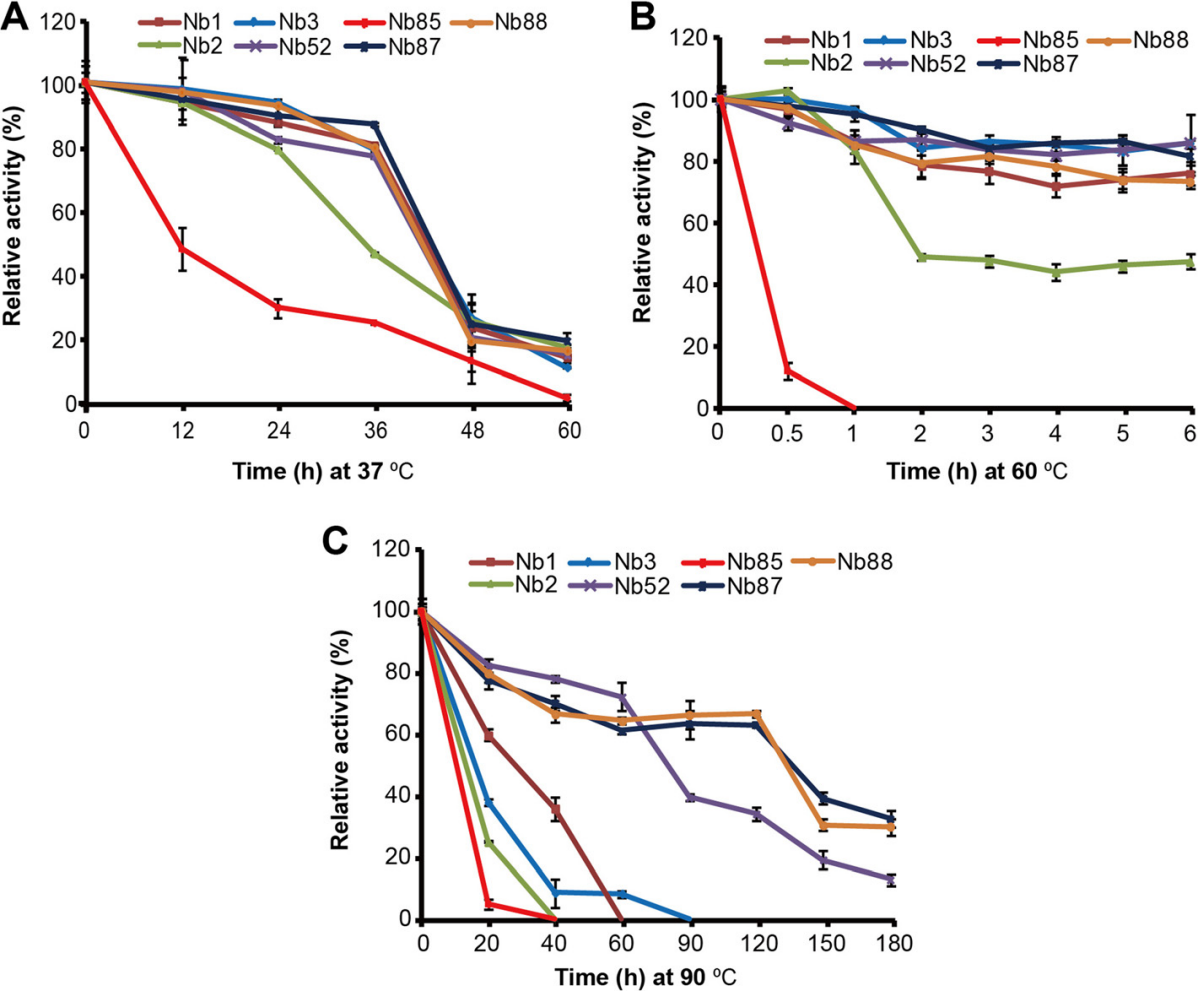
Binding thermostability analysis. The PCT Nbs from the naïve and the immune libraries were incubated at **(A)** 37°C, **(B)** 60°C or **(C)** 90°C for different times to analyze the binding thermostability by ELISA. The activity of the Nbs never been treated was regarded as 100% and three independent experiments were performed.

To further explore whether the naïve library-derived PCT Nbs were as good as the immune library-derived Nbs in thermostability, we performed binding thermostability analysis by ELISA with three PCT specific Nbs derived from the naive library and four target-specific Nbs isolated from the immune library. From the analysis, we found that Nb87 shows the lowest degree of thermal stability, since it could not maintain antigen binding when incubated for 1 h at 60°C or 90°C (Figure 4A, B, C). Among the naïve library-derived PCT Nbs, Nb2 has a worse binding thermostability, but Nb1 and Nb3 could keep binding thermostability profile with three other immune library derived Nbs under the treatment at 37°C and 60°C (Figure 4A, B). However, when the Nbs were treated at 90°C, the immune library-derived Nb52, Nb85 and Nb88 show better binding stability than the three Nbs from the naïve library (Figure 4C). In general, the binding thermostability of Nbs is individually different. According to the analysis, the naïve library-derived PCT Nbs could retain similar binding thermostability as the immune library derived Nbs, at temperatures that were not particularly high. However, when incubated at 90°C, we did not find one Nb from the naïve library that exhibited similar antigen-binding thermostability as the immune library-derived Nbs. It is probably due to the lack of *in vivo* maturation when camels are immunized that the Nbs isolated from the non-immune libraries have a somewhat lower thermal stability. Nevertheless, it should be good enough for most applications, since temperatures above 60°C will not be experienced and also not for prolonged periods.

### Affinity analysis

**Figure 5:**
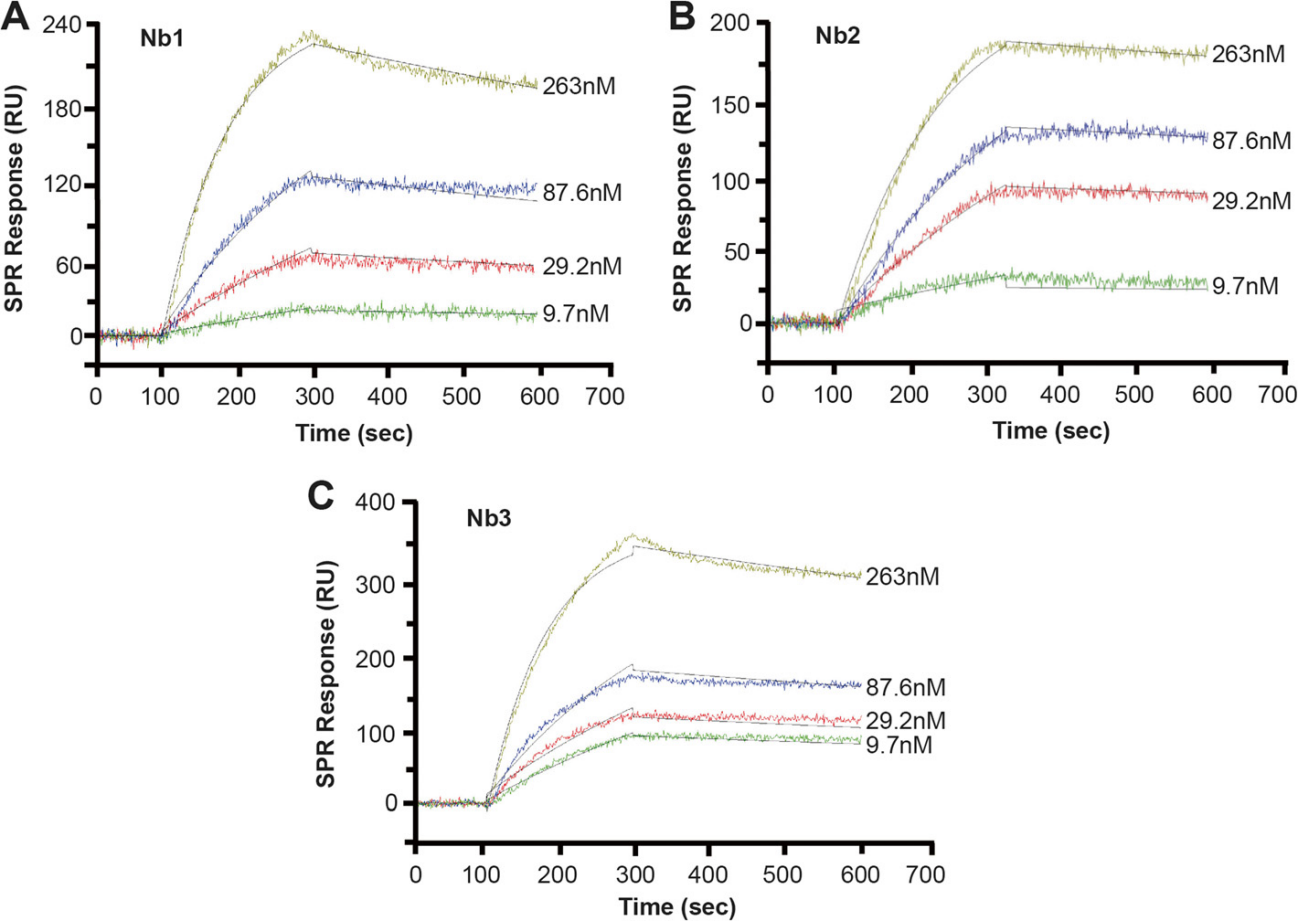
SPRi binding assay. Affinity between PCT and the three Nbs was determined by SPRi binding assay. **(A)** Nb1, **(B)** Nb2 and **(C)** Nb3 were immobilized on the chip surface and PCT in PBST were injected at the concentrations of 9.7, 29.2, 87.6 and 263 nM.

As we wanted to further prove the quality of the three Nbs selected from the naïve library, we measured their affinities by SPRi binding analysis using PlexArray® HT system. As the sensorgrams (Figure 5A, B and C) show, these three Nbs presented their equilibrium dissociation constants (K_D_) ranging from 1.34 ± 0.18 × 10^-9^ to 1.23 ± 0.27 × 10^-8^M (Table 1). Compared to the immune library-derived Nbs, these three Nbs showed similarly high affinity to PCT according to SRP analysis, [23] which provided a great possibility for further antigen detection and diagnostic applications. Overall, the Nbs from the naïve library showed to have excellent affinities, completely comparable to the Nbs derived from the immune library.

### Assessment of Nbs binding to distinct epitopes on PCT and Nb biotinylation

**Figure 6:**
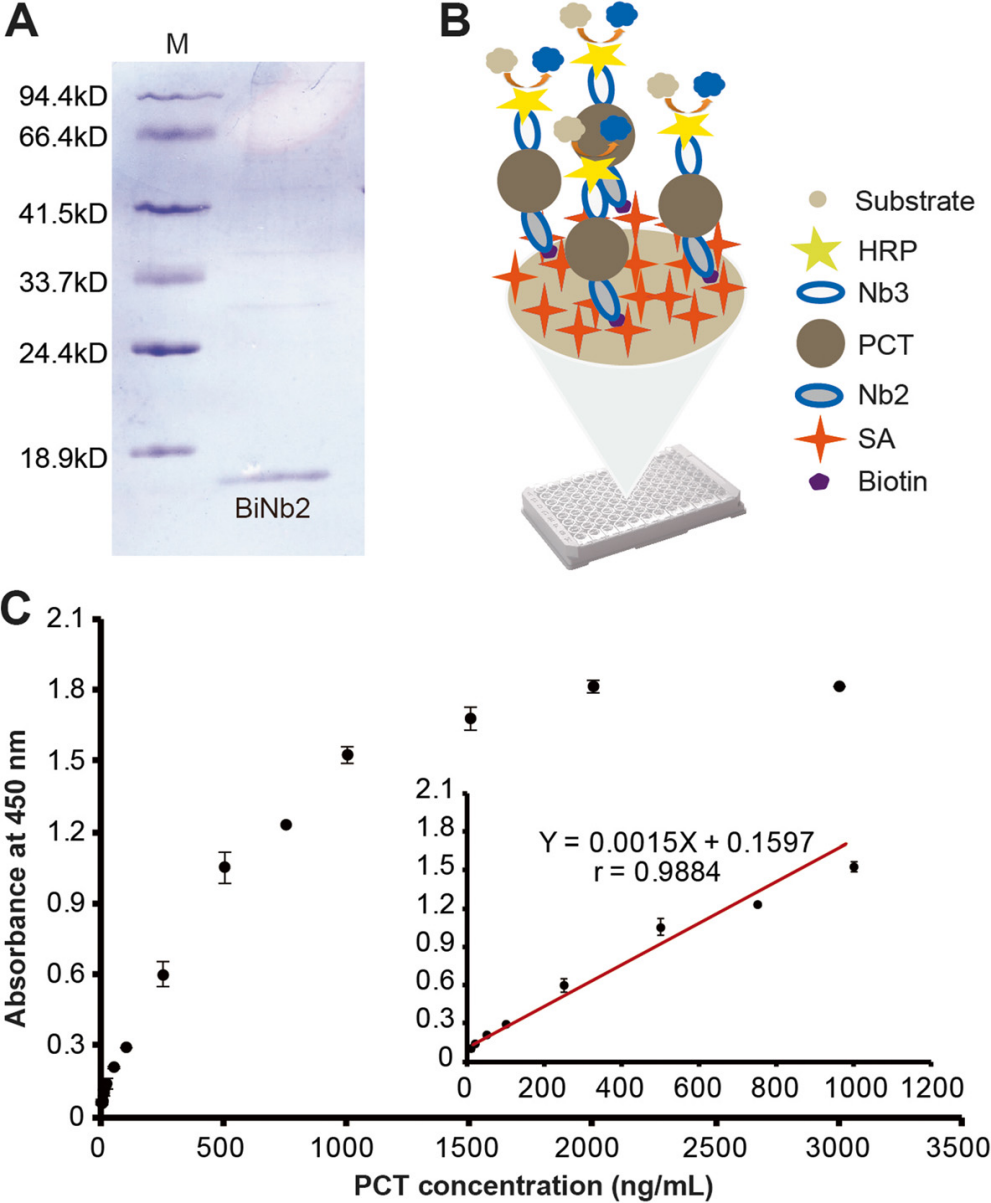
Detection of PCT by the sandwich ELISA based on BiNb2. **(A)** The purified BiNb2 was analyzed by SDS-PAGE. **(B)** Schematic drawing of the proposed sandwich ELISA. **(C)** Calibration curve toward different concentrations of PCT. The linear relationship was in the range from 10 to 1000 ng/mL.

For the further application of the isolated Nbs in PCT detection, we built a biotin-streptavidin (SA) interaction-based ELISA. Therefore, it was necessary to firstly identify two Nbs that recognized two unique epitopes of the same antigen. By coupling the PCT Nbs with HRP and then doing the paired test, among the three Nbs, Nb2 and Nb3 were identified that recognized different epitopes. The Biotin-SA-System is one of the most widely used conjugation pairs in immunoassays, which promotes the analytical sensitivity and can profit from multiple amplification methodologies to even boost the sensitivity. To realize the application of Biotin-SA-System, we needed to modify our Nbs with biotins. In this study, we chose Nb2 to conjugate with biotin as the capture protein and HRP-coupled Nb3 as the chromogenic antibody used in PCT detection. In order to realize Nb2 biotinylation, firstly the VHH genes of Nb2 were sub-cloned into plasmid pBAD17 that contains a biotin acceptor domain (BAD). Afterwards, the recombinant plasmids were co-transformed into WK6 cells with another plasmid pBirA, which can express biotin ligase that catalyzes the ligation between biotin and BAD. BiNb2 was purified by SA Mutein Matrix and analyzed by SDS-PAGE (Figure 6A).

### PCT detection by a sandwich ELISA based on BiNb2

As shown in Figure 6B, BiNb2 was efficiently captured by SA molecules on BeaverNano™ SA Matrix Coated 96-Well Plate as the capture antibody. PCT was recognized and captured by BiNb2, and then Nb3-HRP was used as the detector as it recognized another antigenic site. The absorbance at 450 nm demonstrated a high linearity in the range from 10 to 1000 ng/mL of PCT. The linear equation was calculated as Y = 0.0015 X + 0.1597 with an acceptable linear correlation coefficient (r) of 0.9884 (Figure 6C). Therefore, the Nbs from the naïve library could be functional tools for the determination of disease biomarker.

### Real sample analysis

In order to explore the feasibility for a real sample, the proposed ELISA was used to determine the recoveries of different concentrations (10, 100, 500 and 1000 ng/mL) of PCT in serum samples by standard methods. As shown in Table 2, the recovery was in the range of 93.1-116.6% and the relative standard deviation (RSD) was in the range of 2.42-5.34%. Hence, the developed sandwich ELISA has great potential for clinical application. These results indicated that the Nbs from the proposed naïve library possess practical functions and capabilities, which is crucial for the development of real applications in the future.

**Table 2:** **Detection of PCT in serum samples by the proposed sandwich ELISA**

	**PCT concentration (ng/mL)**			
**Sample**	**Add**	**Found (n = 6)**	**Recovery (%)**	**RSD (%) (n = 6)**
1	10	11.66	116.6	5.34
2	100	93.09	93.1	3.71
3	500	519.09	103.8	4.86
4	1,000	941.87	94.2	2.42

## Conclusions

A large and functional naïve VHH library was constructed, from which, three Nbs families against PCT were successfully isolated. In comparison with the PCT Nbs from an immune library, the naïve library-derived PCT Nbs showed similar affinity and antigen-binding thermostability and thermotolerant sustainability at 37°C and 60°C. Furthermore, two of the PCT Nbs were successfully used to detect PCT based on an improved sandwich ELISA based on the Biotin-SA-System. In brief, such a naïve library can be a powerful and resourceful alternative to the immune libraries. Besides, the characterized Nbs against PCT could provide great values in medical applications.

## Methods

### Materials and instruments

PCT was purchased from Huibiao Biological Technology Co., Ltd. Freund’s adjuvant, horseradish peroxidase (HRP), goat anti-mouse IgG-alkaline phosphatase, Bis (p-nitrophenyl) phosphate (BNPP) and Tetramethylbenzidine (TMB) were purchased from Sigma-Aldrich. Mouse anti-HA tag antibody was obtained from Covance. Fast Track 2.0 Kit and ThermoScript RT-PCR Kit was obtained from InVitrogen. *Pst* I, *Not* I, *Nco* I, *BstE* II and T4 ligase were obtained from NEB (USA). Streptavidin Mutein Matrix was purchased from Roche. 96-well Maxisorp plates were purchased from Thermo Scientific NUNC. DNA markers were provided by Takara. Protein markers were obtained from Vazyme Biotech Co., Ltd and Thermo Scientific. Polyethylene glycol (PEG) 6000 and Biotin were obtained from Shanghai Sangon Biotech. BeaverNano™ Streptavidin Matrix Coated 96-Well Plates were provided by Beaver. Phagemid vector pMECS, VCSM13 helper phages, *Escherichia coli* (*E. coli*) TG1 and WK6 cells, plasmids pBAD17 and pBirA were from Prof. Serge Muyldermans’s lab (Laboratory of Cellular and Molecular Immunology, VUB-Vrije Universiteit, Brussel, Belgium). Bactrian camels were provided by “Joint Center for Nanobody Research & Development between SEU and Egens Bio”. Affinity analysis by surface plasmon resonance imaging (SPRi) binding assay was performed on PlexArray ® HT system (Plexera LLC).

### Naïve VHH library construction

PBLs were isolated from 1 L of fresh blood from twenty four non-immunized bactrian camels. The camels are still alive and are kept on the farm. In the meantime, a spleen was obtained from another non-immunized bactrian camel. All animal experiments were carried out in accordance with the approved guidelines of Southeast University and all experiment protocols were approved by Institutional Ethic Committee of Southeast University.

Total RNA was isolated from the PBLs and the ground spleen sample using a Fast Track 2.0 Kit. The VHH library was constructed according to our previous studies [17,25,26]. Briefly, the cDNA was synthesized by reverse transcription (RT)-polymerase chain reaction (PCR) using a ThermoScript RT-PCR Kit with oligo-dT_12-18_ as the primer. Then a multi-step PCR was used to amplify VHH gene fragments. The first-step PCR was performed with the primers CALL001 and CALL002 [27]. The 600 bp amplicons were extracted from the agarose gel and used as template. The second PCR amplfication was performed with primers VHH-Back and PMCF [28]. Afterwards, the final products were extracted from agarose gel purification. The purified products were ligated into the phagemid pMECS at 16°C for 16 h with T4 DNA ligase after digestion by *Pst* I and *Not* I. Totally, 1680 μg insert DNA and 5600 μg pMECS phagemid were used in ligations and 7000 ligations were performed. For one day’s experiment, 200 ligations were performed. 48 μg insert DNA and 160 μg vector were used and mixed into 20 mL for a ligation mixture. The mixture was then divided into 200 ligations and incubated at 16°C for 16 h. After ligation, we recovered the ligation products and dissolved into 1 mL of ddH_2_O. The ligation products were mixed into 9 mL of fresh TG1 competent cells and divided into 250 electroporations to perform the transformation with the voltage of 1700 V. In total, about 8750 electroporations were performed during the whole library construction. The cells were re-suspended into 100 mL of SOC medium and incubated at 37°C for 1 h after electroporations. Then the cells were re-suspended into 100 mL of LB medium supplemented with 15% (v/v) glycerol and stored at -80°C. After all the electroporations were finished, all the cells stored at -80°C were slowly thawed, mixed, centrifuged and re-suspended into 70 mL of LB medium. From which, 500 μL of the cells was used for the identification of library size. Another 69 mL of the cells were diluted with LB medium and were plated onto plates containing solid LB medium supplemented with 100 μg/mL ampicillin and 2% (w/v) glucose, and cultured at 37°C overnight. Finally, the colonies were scraped into 300 mL of liquid LB medium supplemented with 15% (v/v) glycerol and were stored at -80°C.

### Selection of PCT Nbs

Based on the successful construction of the naïve library, we used PCT as the target protein to isolate the antigen binders. We only took a smaller sample of the library to investigate whether good quality of binders can be isolated. It is obvious that small percent of library may lose the diversity of Nbs during selection, which was also reported in a previous study [24]. About 1 × 10^10^ TG cells from the library stock were grown in 2 × TY medium containing 100 μg/mL ampicillin and 1% (w/v) glucose at 37°C. After 2 h, the cells were infected with 1 × 10^12^ VCSM13 helper phages for 30 min at room temperature (RT). The infected cells were harvested and resuspended into 2 × TY medium supplemented with 100 μg/mL ampicillin and 70 μg/mL kanamycin, then the cells were incubated overnight at 37°C and 220 rpm. The phages were precipitated from culture supernatant with PEG 6000/NaCl and resuspended in sterile PBS. About 2 × 10^11^ phage particles were used for each bio-panning against PCT (200 μg/mL) coated on 96-well Maxisorp plates. After four consecutive rounds of bio-panning, 95 individual colonies were randomly chosen and each was grown in 1 mL of terrific broth (TB) medium. The expression of Nbs was induced with 1 mM isopropyl β-D-1-thiogalactopyranoside (IPTG) overnight at 28°C. Positive clones expressing anti-PCT Nbs were identified by PE-ELISA. Then the identified clones were sent for sequencing. The amino acids sequences of Nbs genes were analyzed and classified into different families based on their sequence diversity in CDR3 regions.

### Expression and purification of PCT Nbs

The plasmids of the different Nb families were extracted from TG1 cells and electro-transformed into *E. coli* WK6 cells. The WK6 cells were cultured in 330 mL of TB medium supplemented with 0.1% (w/v) glucose, 100 μg/mL ampicillin and 2 mM MgCl_2_ till an OD_600_ of 0.6, and induced with 1 mM IPTG overnight at 28°C. The periplasmic proteins were released by osmotic shock from the cells. The PCT-specific Nbs were produced as C-terminal hexahistidine (His_6_)-tag proteins and purified with 500 mM imidazole solution. Afterwards, the Nbs were ultra-filtrated to remove the imidazole molecules and dialyzed into PBS solution. Ultimately, the purified PCT-specific Nbs were diluted to 1 mg/mL as the stock and analyzed by sodium dodecyl sulfate polyacrylamide gel electrophoresis (SDS-PAGE).

### PCT-binding thermostability analysis

Heat resistance of the naïve library-derived PCT Nbs and the immune library-derived PCT Nbs were assessed by incubating them at 37°C for various times (0, 12, 24, 48, 60 and 72 h), at 60°C for 0.5, 1, 2, 3, 4, 5 and 6 h, and at 90°C for 20, 40, 60, 90, 120,150 and 180 min. 96-well Maxisorp plates were coated with 100 μL of 5 μg/mL PCT in coating buffer (0.1 M NaHCO_3_, pH 9.6), and the corresponding blank controls were set. After blocking with 1% (w/v) skim milk for 2 h, 100 μL of Nbs solution (10 μg/mL) were added into the wells to incubate for 1 h. After washing, mouse anti-HA tag antibody was added for 1 h followed by goat anti-mouse IgG-alkaline phosphatase for 1 h at RT. After 5 times of washing with PBST (PBS with 0.05% tween-20), BNPP substrate solution was added and finally the plates were read using Bio-Rad iMark^TM^ (Bio-Rad, USA) at 405 nm.

### Affinity analysis by SPRi binding assay

SPRi binding assay was performed according to our previous study [17]. Briefly, the Nbs were spotted on the NanoCapture 3D-chip surface for 1 h to immobilize. Afterwards, the chip was incubated in 2 mL of 1 M ethanolamine-HCl (pH 8.5) for 20 min in order to absolutely block the remaining activated NHS groups. The chip was loaded into PlexArray® HT, and flew PBST as running buffer at a constant flow rate for setting up assay. 4 series of PCT dilutions (9.7, 29.2, 87.6 and 263.0 nM) were sequentially injected at a flow rate of 2 μL/s. Finally, in order to elute the bound analytes for returning to baseline value, the chip was then regenerated by 1:200 (v/v) H_3_PO_4_ at a flow rate of 3 μL/s. The whole assays were conducted at the same conditions as during the set up. All binding data were analyzed using Plexera Data Analysis Module. Binding curves were fitted with 1:1 Langmuir binding model for a binding kinetics.

### HRP coupling

In order to determine whether the isolated PCT Nbs could recognize different epitopes of PCT, HRP was used to couple to the purified Nbs firstly as described in our previous study [25]. Briefly, 100 μL of fresh NaIO4 (0.1 M) was incubated with 200 μL of 5 mg/mL HRP solution for 30 min at 4°C. Next, 100 μL of 2.5% (v/v) ethylene glycol were added and incubated for 30 min at RT. Then 1 mL of PCT Nbs (1 mg/mL) was added and the mixture was incubated at 4°C overnight in the dark. Afterwards, 20 μL of NaBH_4_ (5 mg/mL) were mixed into the PCT/HRP mixture for 3 h at 4°C. Finally the mixture was transferred to an Ultra-filtration column to replace the buffer with PBS.

### Assessment of Nbs binding to distinct epitopes on PCT

In order to identify two Nbs that can recognize distinct epitopes on PCT, an indirect ELISA was performed. A 96-well Maxisorp plate was coated with 5 μg/mL Nbs in 0.1 M NaHCO_3_ (pH 9.6) at 4°C overnight. After washing with PBST and blocking with 3% (w/v) bovine serum albumin (BSA), 100 μL of PCT (2 μg/mL) was added to the wells and PBS was added to the corresponding wells as the control for 1 h. The HRP-conjugated Nbs (2 μg/mL) were added to the wells. After several times of washing by PBST, 100 μL of TMB solution was added and incubated for 5-10 min at RT. The enzyme reaction was stopped by 50 μL of 2 M H_2_SO_4_ and the absorbance was read at 450 nm.

### Nb biotinylation *in vivo*

According to the results of epitope mapping, we chose PCT Nb2 to conjugate with biotin. The biotinylation was performed as our previous studies described [17,25]. Briefly, the gene encoding PCT Nb2 was sub-cloned into plasmid pBAD17 and the recombinant plasmid was co-electrotransformed into competent WK6 cells with the plasmid pBirA. The cells were cultured in 330 mL of TB medium and 330 μL of 50 mM biotin was added when the OD was increased to about 0.4. The expression of proteins was induced by 1 mM IPTG overnight at 28°C. The biotinylated PCT Nb2 (BiNb2) was purified by SA Mutein Matrix with elution buffer containing 4 mM biotin. The residual biotin molecules were removed by Ultra-filtration column with PBS for several times.

### PCT detection by a sandwich ELISA based on BiNb2

100 μL of BiNb2 (2 μg/mL) in PBST was added per well of a BeaverNano™ SA Matrix Coated 96-Well Plate at RT for 1.5 h. After 5 times of washing with PBST and blocking with 5% BSA in PBST for 1 h, the wells were incubated with serial dilutions (0, 1, 2, 5, 10, 20, 50, 100, 250, 500, 750, 1000, 1500, 2000 and 3000 ng/mL) of PCT for 1 h. Afterwards, 100 μL of PCT Nb3 conjugated to HRP (Nb3-HRP) (1 μg/mL) in PBST containing 5% BSA was added. After 1 h, 200 μL of PBST was added to the wells, and the plate was shaken for 5 min at RT. 10 times of washing were repeated and 100 μL of TMB solution was added to incubate for 10 min at RT. Finally, the absorbance was read at 450 nm after the enzyme reaction was stopped by addition of 50 μL of 2 M H_2_SO_4_ solution.

## Additional file

Additional file 1:
**Supplemental data for this article can be accessed on the publisher’s website.**

## Abbreviations

Nb: Nanobody
Nbs: Nanobodies
VHH: Variable domain of the heavy chain of the heavy-chain only antibody
PCT: Procalcitonin
IgG: Immunoglobulin G
SDS-PAGE: Sodium dodecyl sulfate polyacrylamide gel electrophoresis
Fabs: antigen-binding fragments
scFvs: Single-chain antibody fragments
HCAbs: Heavy chain-only antibodies
PBLs: Peripheral blood lymphocytes
CFU: Colony-forming units
CDR: Complementarity determining region
BSA: Bovine serum albumin
*E. coli*: *Escherichia coli*
HRP: Horseradish peroxidase
ELISA: Enzyme-linked immunosorbent assay
SRPi: Surface plasmon resonance imaging
IPTG: Isopropyl-β-d-thiogalactoside
His_6_: hexahistidine
HA: Hemagglutinin
pH: Potential of hydrogen
PE-ELISA: Periplasmic extract ELISA
BNPP: Bis (p-nitrophenyl) phosphate
TMB: 3, 3’, 5, 5’-Tetramethylbenzidine
SA: Streptavidin
TB: Terrific broth
