# Supplementary material for: Characterization and applications of Nanobodies against human procalcitonin selected from a novel naïve Nanobody phage display library
Source: J Nanobiotechnology. 2015 May 6;13:33. doi: 10.1186/s12951-015-0091-7 (PMC4475299; doi:10.1186/s12951-015-0091-7)
Supplement: Additional file 1: — Supplemental data for this article can be accessed on the publisher’s website. [file 12951_2015_91_MOESM1_ESM.docx]

**Characterization and applications of Nanobodies against human procalcitonin selected from a novel naive Nanobody phage display library**

Junrong Yan^1^, Pingyan Wang^1^, Min Zhu^1^, Guanghui Li^1^, Ema Romão^4^, Sheng Xiong^3*^, Yakun Wan^1,2*^

^1^The Key Laboratory of Developmental Genes and Human Disease, Ministry of Education, Institute of Life Sciences, Southeast University, Nanjing 210096, PR China

^2^Jiangsu Nanobody Engineering and Research Center, Nantong 226010, PR China

^3^Institute of Biomedicine & National Engineering Research Center of Genetic Medicine, College of Life Science and Technology, Jinan University, Guangzhou 510630, PR China.

^4^Laboratory of Cellular and Molecular Immunology, Vrije Universiteit Brussel, Faculty of Science, Pleinlaan 2, 1050 Brussels, Belgium

**Corresponding author*:**

Yakun Wan, PhD, Professor. Address: Sipailou No.2, Southeast University, Nanjing, PR China. Tel.: 86-25-83790967. Fax: 86-25-83790960. Email: [ywansystemsbiology@gmail.com](mailto:ywansystemsbiology@gmail.com).

Sheng Xiong, PhD. Address: Room 730, 2nd Building of Sci & Tech, 601W Huangpu Ave, Guangzhou, PR China. Tel.:+86-20-85220504. Fax: +86-20-85220504. Email: xiongsheng@jnu.edu.cn.

Fig. S1

**
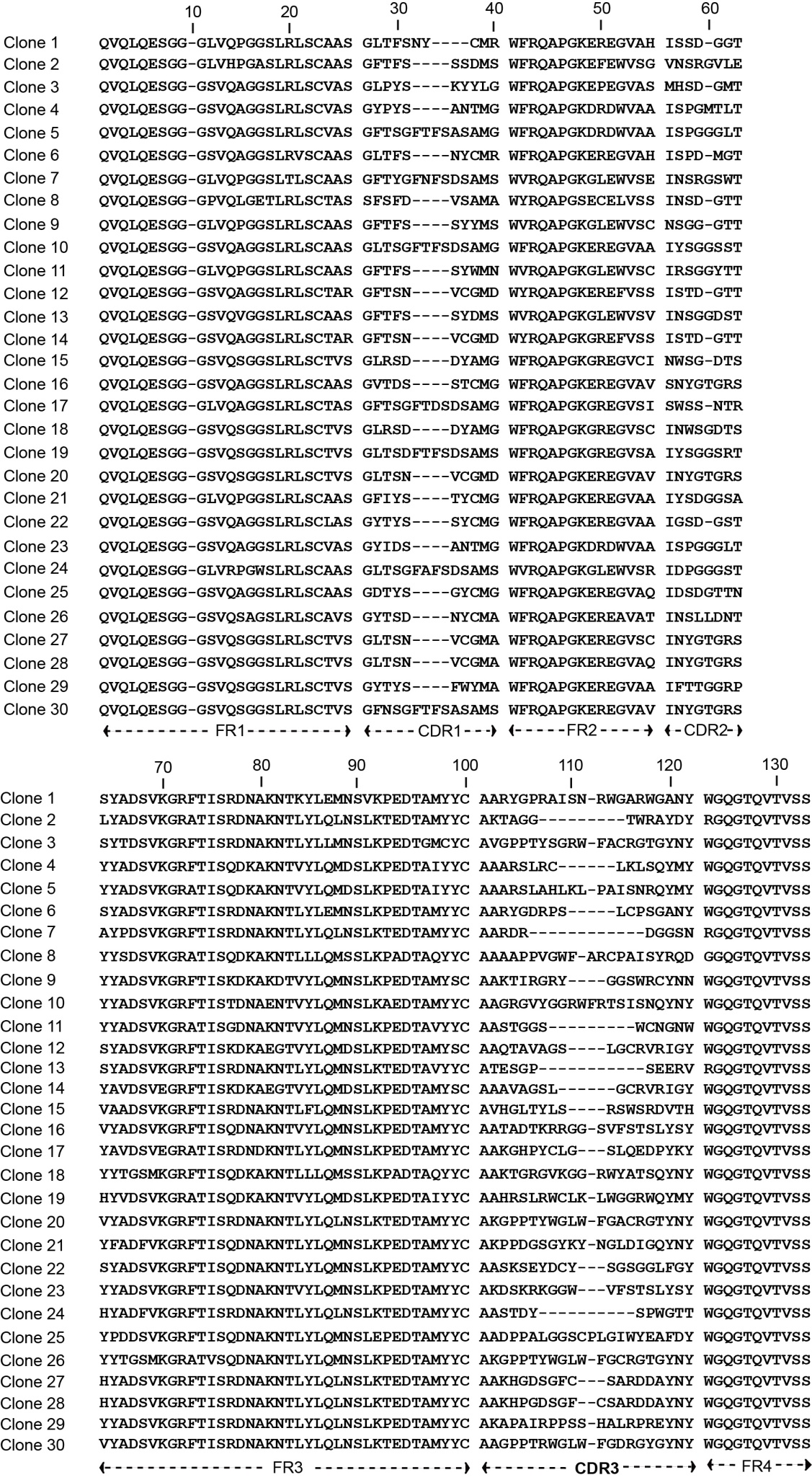
**

Fig. S2

**
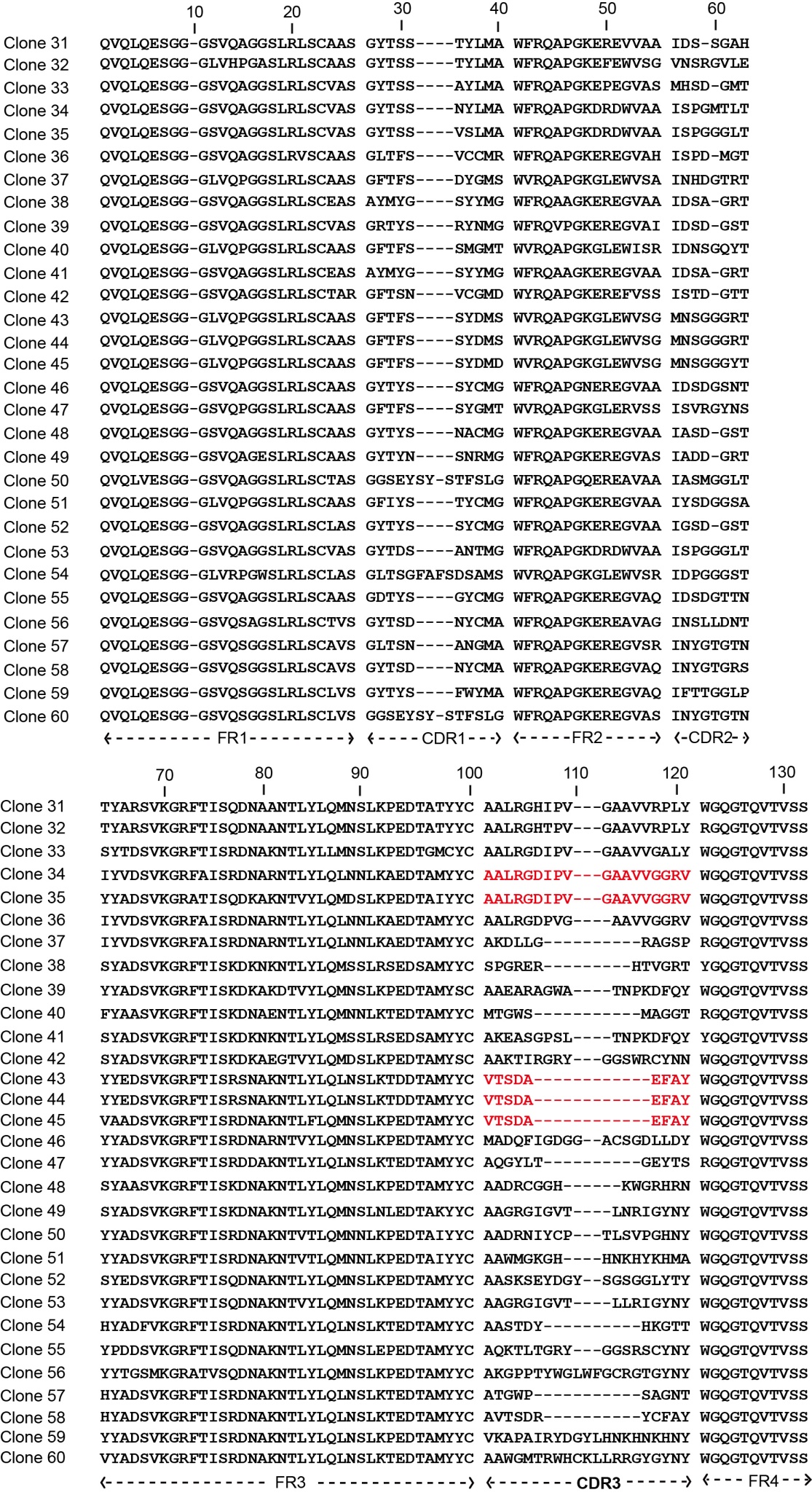
**

Fig. S3


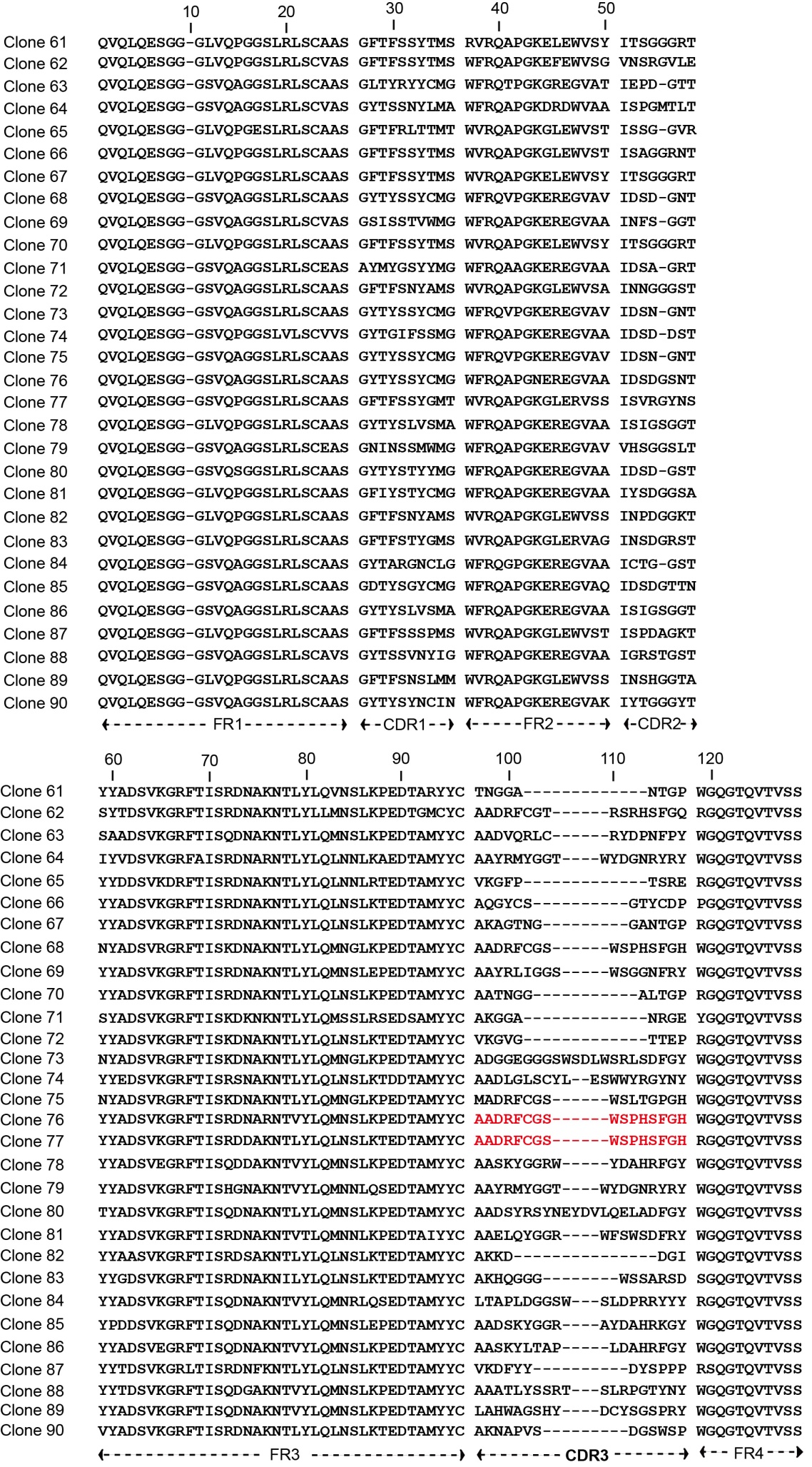


Fig. S4

**
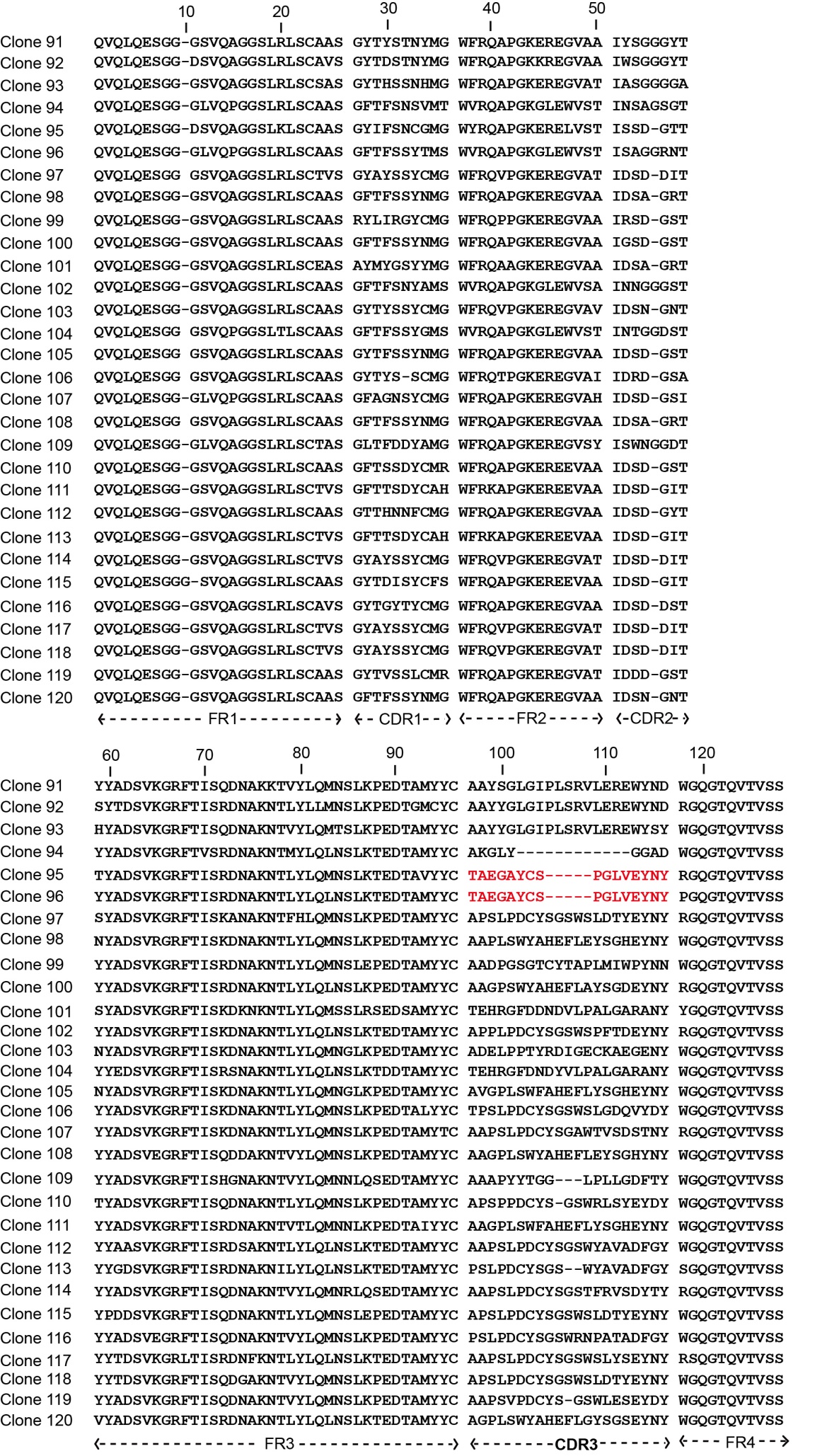
**

Fig. S5


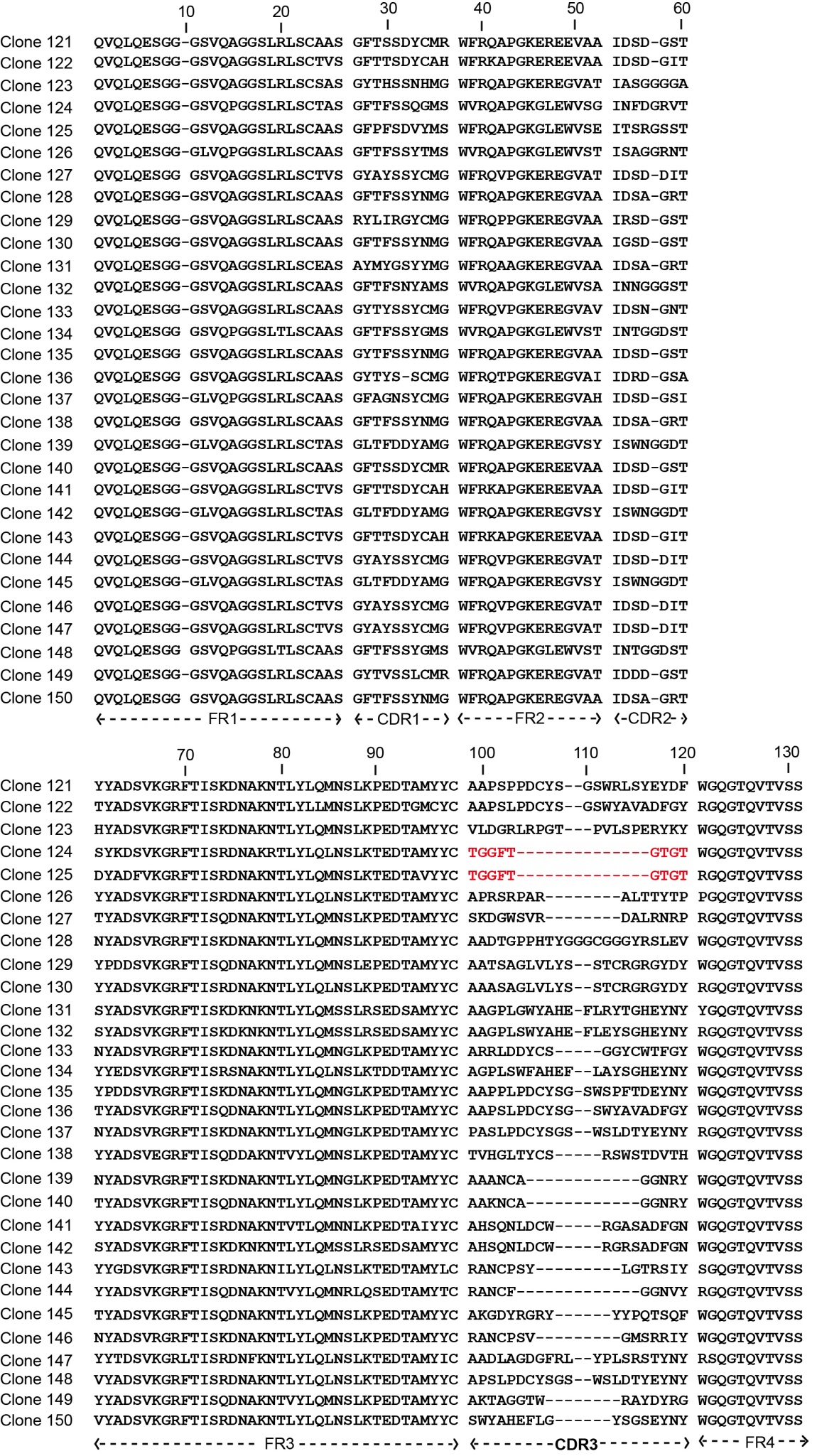


Fig. S6


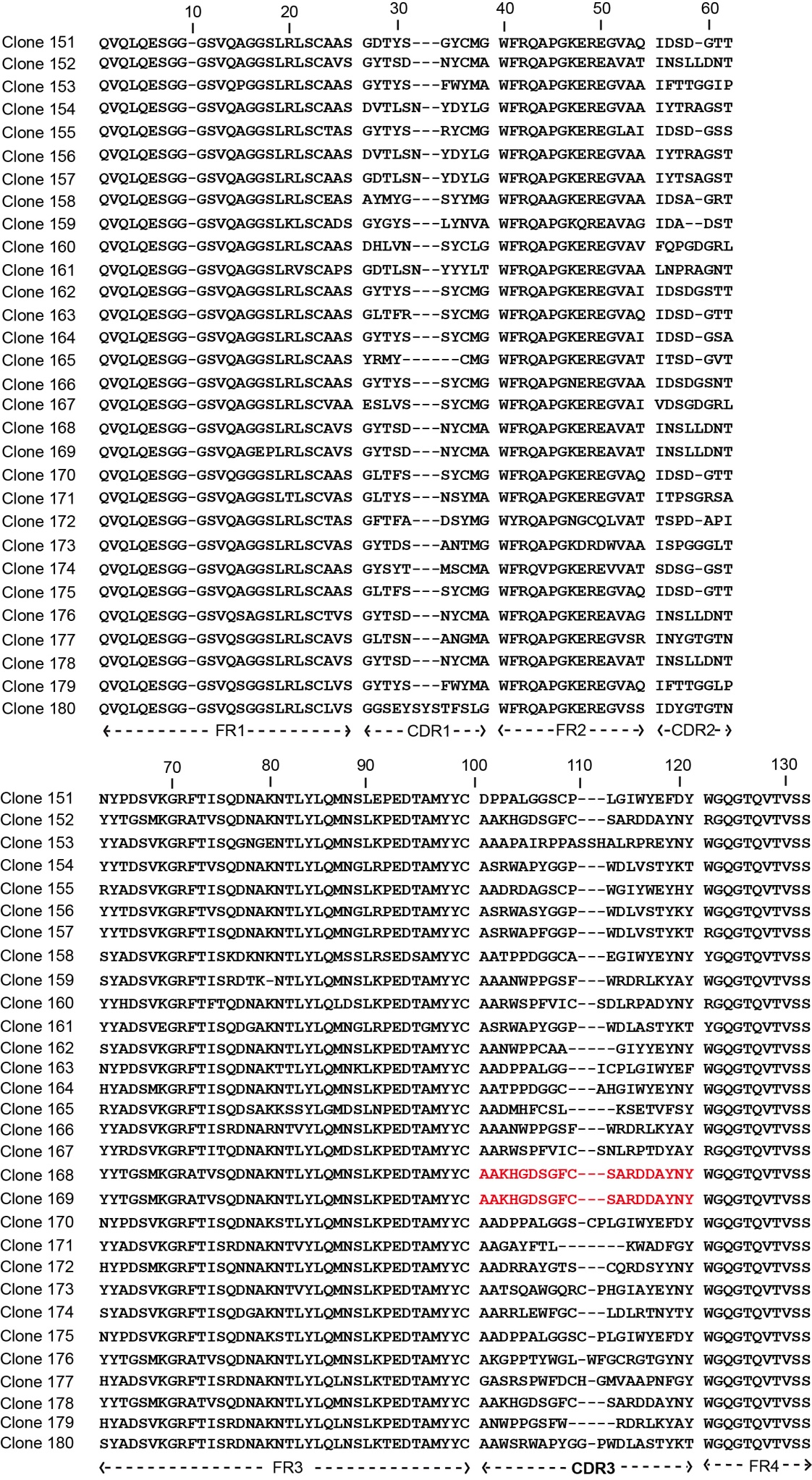


Fig. S7


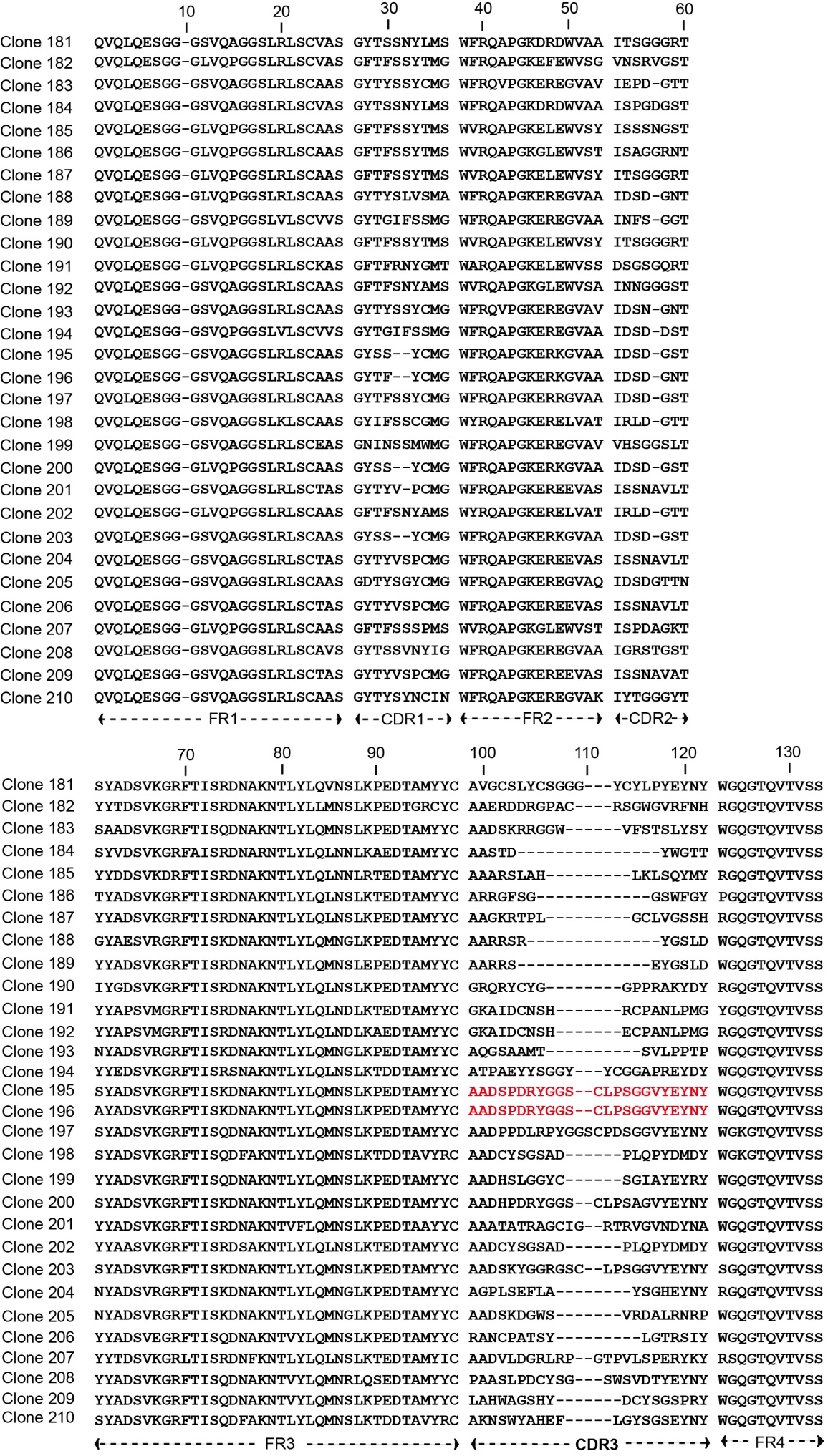


Fig. S8


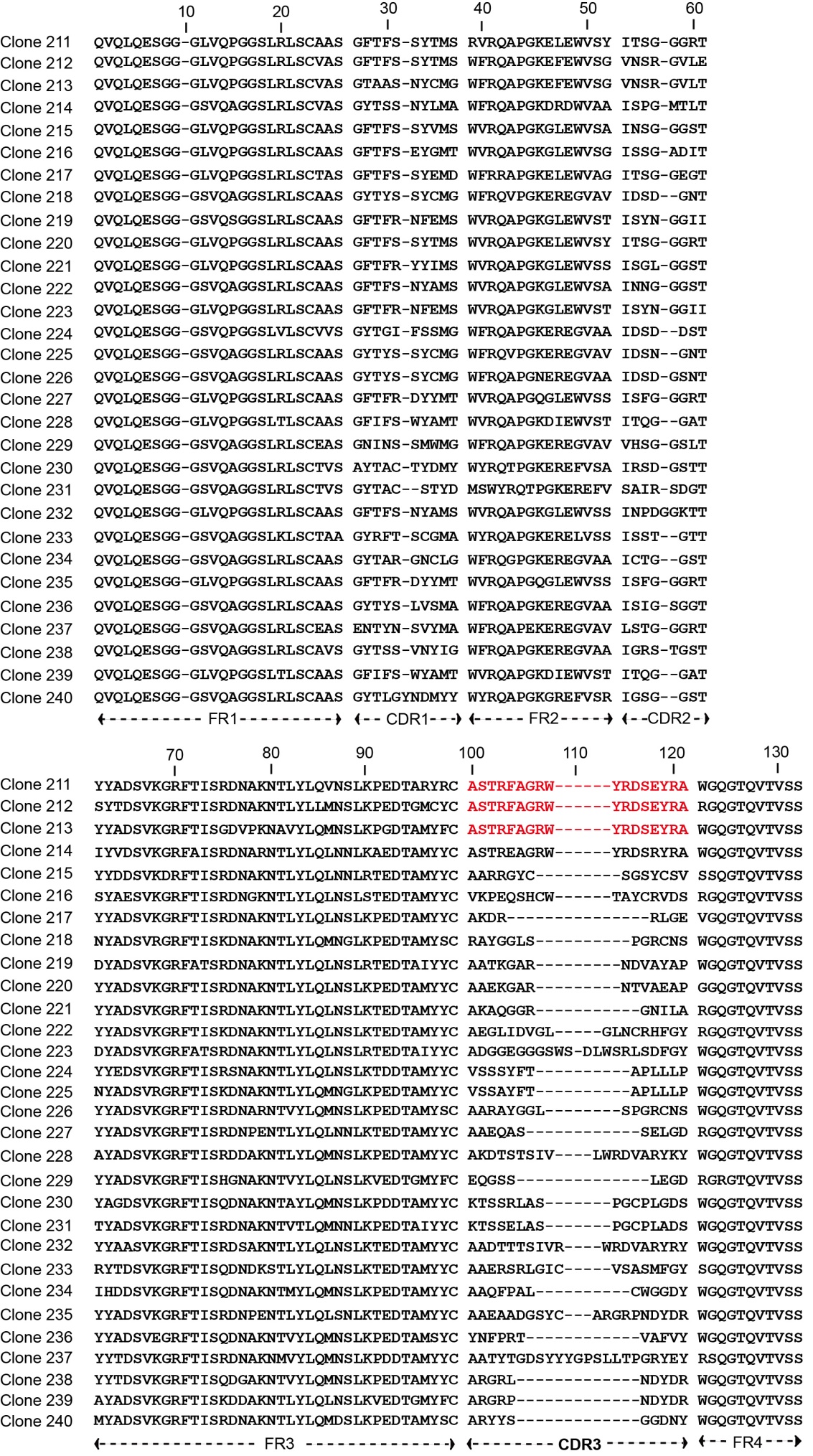


Fig. S9


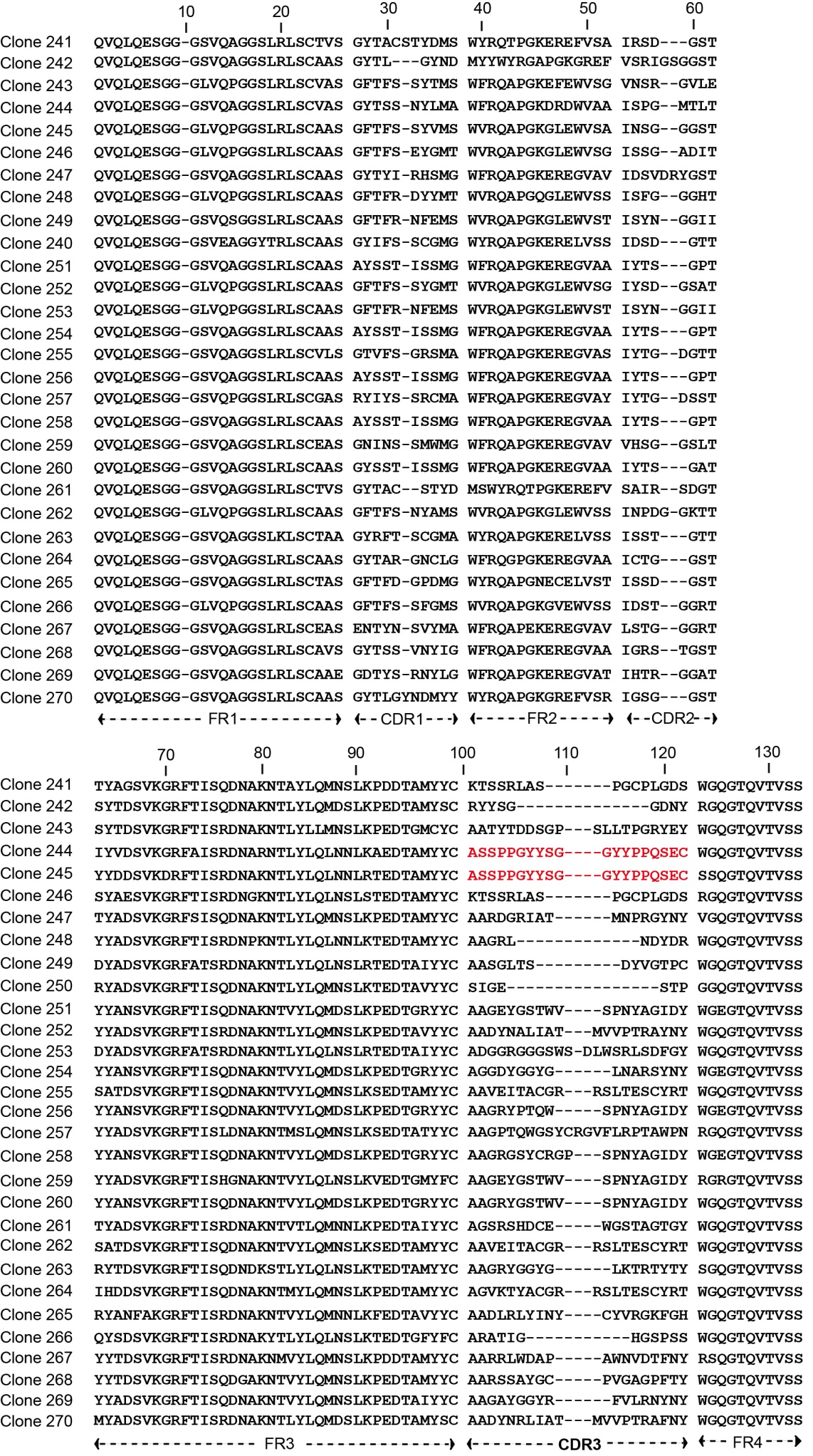


Fig. S10


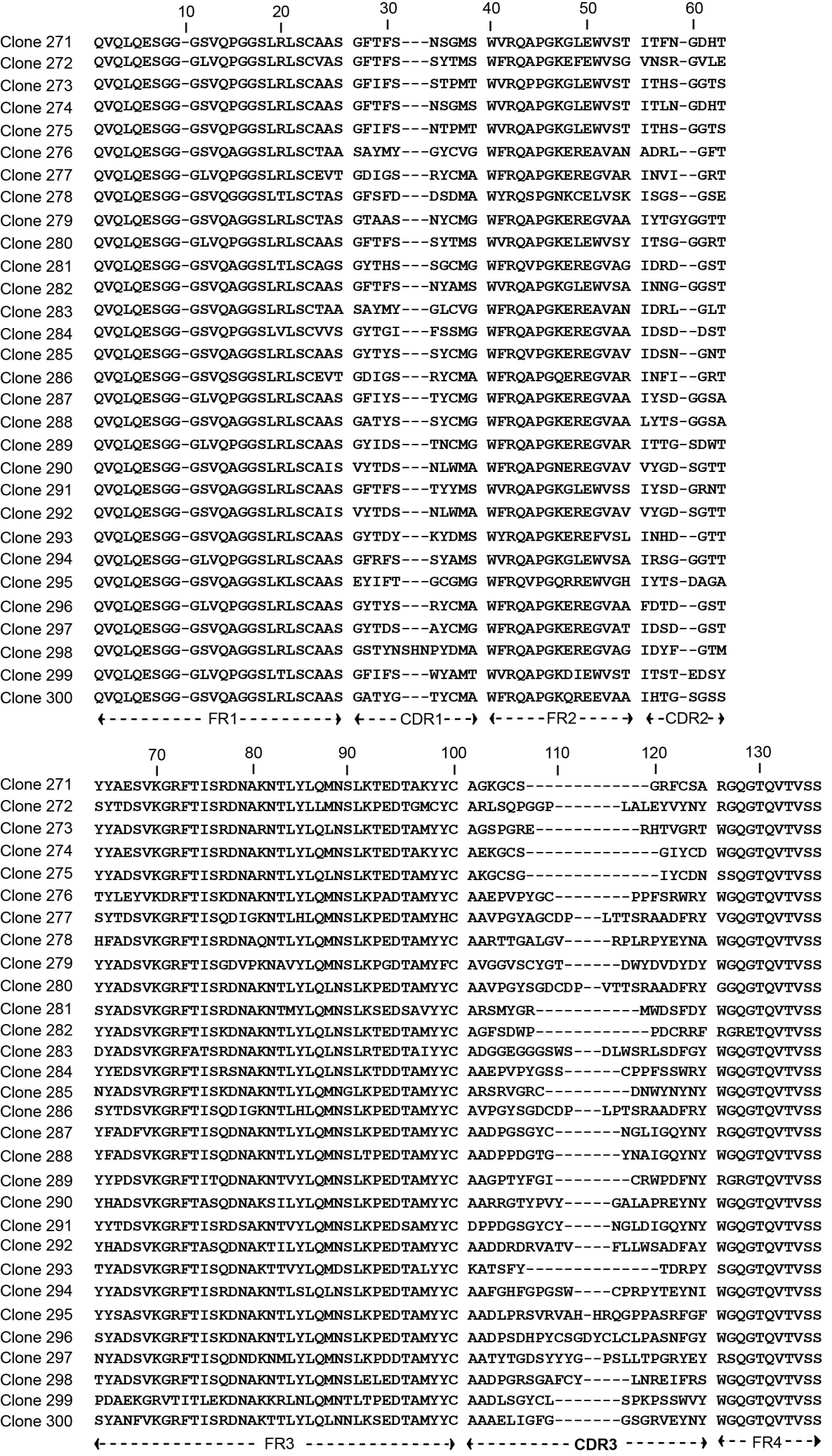


**Fig. S1-S10.** **Diversity determination of the naïve library.** 300 individual clones were randomly chosen for sequencing to determine the library diversity. Amino acids positions of the framework FRs and the three antigen-binding loops (CDR1, CDR2 and CDR3) are numbered according to the IMGT Scientific chart for the V-Domain and are indicated at bottom. Clones exhibited the same amino acids in CDR3 are colored in red in CDR3.
